# Rising Obesity in Malaysia (1990–2023): A Comprehensive Analysis of Temporal Trends, Sex Inequalities, and Lifestyle Drivers 

**DOI:** 10.3389/phrs.2026.1609497

**Published:** 2026-01-30

**Authors:** Vetriselvan Subramaniyan

**Affiliations:** Sir Jeffrey Cheah Sunway Medical School, Faculty of Medical and Life Sciences, Sunway University, Bandar Sunway, Malaysia

**Keywords:** adult obesity trends, lifestyle and urbanization factors, obesogenic transition, public health impact, sex and ethnic disparities

From 1990 to 2023, Malaysia transitioned from a low to moderate adult obesity prevalence to a high prevalence, highlighting a significant public health burden. In 1990, the WHO/NCD-RisC age-standardized estimate for adult obesity was approximately 8.4% [1]. By 2000, obesity had escalated, with NCD-RisC/WHO trend estimations indicating Malaysia’s prevalence in the low-teens percentage range at the century’s turn. By 2010, estimates had grown to the mid-teens, demonstrating consistent rises throughout the 1990s and 2000s, attributed to urbanization and dietary modifications. National surveys indicate an increasing prevalence of overweight and obesity throughout the 2010s. The NHMS 2011 documented an adult combined prevalence of overweight and obesity in the mid-40% range, as indicated by NHMS infographics and the 2011 prevalence research, which report significant obesity rates by sex [2]. In 2019, national NHMS statistics indicated that approximately 50.1% of adults were overweight or obese, with 19.7% categorized as obese (15.3% of males and 24.7% of women). The most recent NHMS 2023 revealed that the prevalence of combined overweight and obesity increased to 54.4% (overweight approximately 32.6%, obese approximately 21.8%), along with ongoing increases in abdominal obesity; WHO age-standardized data indicate an overall adult obesity estimate of approximately 17.9% (2022), aligning with this increasing trajectory [3].

Sex and lifestyle disparities are significant and consistent: females have consistently exhibited a higher prevalence of obesity than males in national surveys (e.g., NHMS 2019 and other studies: women ≈24–21% vs. men ≈15–16% depending on the year), and cohort analyses indicate more pronounced increases in BMI and waist circumference among women and certain ethnic groups. The NHMS 2023 identifies significant lifestyle factors such as elevated sedentary behavior, inadequate physical activity (with numerous adults reporting insufficient exercise, minimal active transportation, and low engagement in recreational physical activities), poor sleep habits, and dietary shifts towards energy-dense processed foods—all associated with the increasing prevalence of overweight and obesity. Ethnicity and urban residency are significant factors: Malays and Indians typically exhibit a greater prevalence of obesity compared to Chinese individuals in various studies, whereas urban populations generally demonstrate elevated rates of overweight, obesity, and abdominal obesity relative to rural populations [4].

**FIGURE 1:**
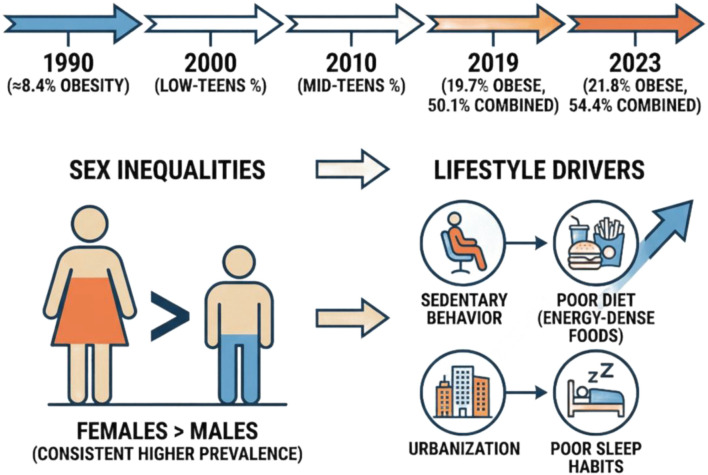
Trends in adult obesity in Malaysia (1990–2023).

As shown in Figure 1, Malaysia’s adult obesity rate nearly doubled from the 1990 baseline to the 2010s–2020s, with the latest national survey (NHMS 2023) indicating approximately 21.8% of the population is obese and 54.4% is overweight or obese overall. Women, specific ethnic groups (Malay, Indian), and sedentary individuals are disproportionately impacted, reflecting a pervasive, population-wide obesogenic transition influenced by dietary habits, physical activity, and demographic changes [3].

These prolonged patterns highlight a persistent obesogenic transition in Malaysia that currently jeopardizes health system resilience, worker productivity, and healthy aging. The enduring nature of sexual and ethnic disparities indicates that standardized interventions will be inadequate, necessitating the immediate implementation of culturally specific, gender-responsive solutions. Increasing abdominal obesity indicates elevated future risks of diabetes, cardiovascular disease, and metabolic diseases. The robust connections between sedentary lifestyles, urban habitation, and nutritional changes underscore the necessity for cohesive policies including urban planning, food systems, transportation, and occupational health. In the absence of effective, comprehensive preventative strategies, obesity-related health issues and healthcare expenditures are expected to increase significantly in the forthcoming decades.
