# Supplementary material for: Rising Obesity in Malaysia (1990–2023): A Comprehensive Analysis of Temporal Trends, Sex Inequalities, and Lifestyle Drivers
Source: Public Health Rev. 2026 Jan 30;47:1609497. doi: 10.3389/phrs.2026.1609497 (PMC12900780; doi:10.3389/phrs.2026.1609497)
Supplement: Supplementary file 1 [file DataSheet1.docx]

**
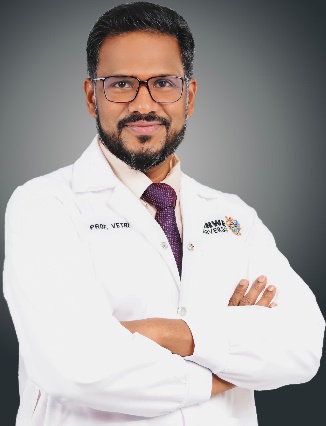
Author Profile**

Vetriselvan Subramaniyan is a pharmacology professor at Sunway University in Malaysia. He has been involved in research, teaching, and academic leadership for more than 16 years. With a focus on translational pharmacology, obesity epidemiology, and evidence-based approaches to treat obesity-related non-communicable diseases, his research focuses on obesity, metabolic disorders, and public health. He has obtained competitive research funding, including national grants and a Monash University Research Travel Grant, to foster international collaboration and high-impact translational research. Stanford University ranks him in the Top 2% of Scientists in the World.
